# Precious metals recovery from aqueous solutions using a new adsorbent material

**DOI:** 10.1038/s41598-021-81680-z

**Published:** 2021-01-21

**Authors:** Oana Grad, Mihaela Ciopec, Adina Negrea, Narcis Duțeanu, Gabriela Vlase, Petru Negrea, Camelia Dumitrescu, Titus Vlase, Raluca Vodă

**Affiliations:** 1 Faculty of Industrial Chemistry and Environmental Engineering, Polytechnic University of Timişoara, Victoriei Square, No. 2, 300006 Timisoara, Romania; 2 Research Centre for Thermal Analysis in Environmental Problems, West University of Timisoara, Pestalozzi Street 16, 300115 Timisoara, Romania

**Keywords:** Pollution remediation, Polymers, Chemical engineering

## Abstract

Platinum group metals (PGMs) palladium, platinum, and ruthenium represent the key materials for automotive exhaust gas treatment. Since there are no adequate alternatives, the importance of these metals for the automotive industry is steadily rising. The high value of PGMs in spent catalysts justifies their recycling. Therefore, it is really important to recovery platinum group metals from aqueous solutions. Of the many PGMs recovery procedures, adsorption is a process with a good efficiency, but an important role is played by the adsorbent material used into the process. In order to improve the adsorption properties of materials were developed new methods for chemical modification of the solid supports, through functionalization with different extractants. In present paper a new adsorbent material (Chitosan-DB18C6) was used for PGMs recovery. The new adsorbent material was produced by impregnating Chitosan with dibenzo-18-crown-6-ether using Solvent Impregnated Resin (SIR) method. The crown ethers were chosen as extractant due to their known ability to bind metallic ions, whether they are symmetrically or unsymmetrically substituted. In order to determine the PGMs recovery efficiency for new prepared adsorbent material the equilibrium and kinetic studies were performed. Also, to study the PGMs adsorption mechanism the experimental data were modelled using pseudo-first-order and pseudo-second order kinetic models. Experimental data were fitted with three equilibrium isotherm models: Langmuir, Freundlich and Sips. The results proved that new adsorbent material (Chitosan-DB18C6) is an efficient adsorbent for PGMs recovery from aqueous solutions.

## Introduction

Platinic metals group (PGMs) it is a group containing 6 chemical elements which have similar physical and chemical properties. This group is also divided into three different subgroups: iridium, platinum (IPGEs and palladium (PPGEs) groups^1^. Due to their specific chemical and physical properties platinic group metals (such as good corrosion resistance, high melting point, good catalytic properties)^2^ are widely used in different industries: electronic industry, catalysts in oil processing industry, medical field and jewelry production^3^.

In this moment around 90% from the entire quantity of platinic metals comes from two countries – Russia and South Africa, rest of the world being dependent on imports^2^. High cost of PGMs is related with the diminishing availability of the natural resources concomitant with the difficulties related to the mining of such elements^4,5^. Therefore, from viewpoint of full utilization of resources it is quite important the effective recovery of PGM’s metals from both natural ore and industrial waste. Recovery of such metals from spent systems can represent an efficient way to reduce the dependence on this limited raw material^2,6,7^.

For effective recovery were developed several methods, such as: solvent extraction^8^, ion exchange^9^, membrane separation^2,10^, complexation^11^, retention on porous polymers^12^, coprecipitation, sequential distillation, precipitation and adsorption which is presented in the literature as the method with higher efficiency for recovery of PGM’s from aqueous solutions^2,4,5,11,13–15^.

From all these recovery methods, adsorption seems to be the most suitable method for the recovery of PGM’s from diluted solutions, due to low cost and high efficiency^14,16–19^. Based on technical and economical points of view a special attention is granted to the materials with good adsorbent properties. From the cost as well as technical perspective, during the last decade, chitosan was preferred for removal and recovery of precious metallic ions^13,16^.

In the current stage of development are selected the materials with good adsorbent properties which represent a good technique for removal of PGM’s from solutions with low concentrations due to low cost and higher efficiency. In this context where used adsorbent materials such as: chitosan^20^, iron oxide^21^, activated graphite^22^, zeolites^23^, and adsorbent materials obtained by synthesis or functionalization with pendant groups containing N, S or P atoms, having selective properties for these groups of metals^3,24–27^.

Aim of present study was the synthesis, followed by the test of adsorptive properties of new materials obtained by functionalization of chitosan with crown ethers. It is well known that chitosan is a linear polysaccharide formed from fragments of glucosamine (deacetylate unit) and N-acetyl-glucosamine (acetylated unit) randomly distributed, being a non-toxic adsorbent used for removal of metallic ions^28^. Presence of N into the chitosan structure leads at the improvement of adsorbent properties of metallic ions by electrostatic attractions^19^. Some limitation appears because of his solubility into the acidic media. In order to overcome this limitation, the structure of chitosan can by modified by functionalization with different pendant groups, leading at improvement of its adsorbent properties for PGM’s ions.

One possible compound used for functionalization of chitosan are crown ethers which represent a macrocyclic polyether organic compound. It is known that the crown ethers can easily link metallic ions forming complex compounds.

In present work the chitosan adsorbent has been functionalized with dibenzo-18-crown ether 6 (DB18C6). During functionalization process the N atoms from chitosan structures form new hydrogen bonds with the oxygen atoms from crown ether leading at a new complex adsorbent which present a higher selectivity for metallic ions. Functionalization process was carried out by using solvent impregnated resin (SIR) method, and further used for recovery of Pd(II), Pt(IV) and Ru(III) by adsorption^20,21^. Also was evidenced the higher affinity of adsorbent material for one of these ions.

## Materials and methods

### Adsorbent synthesis and characterization

In order to prepare a new adsorbent material by functionalization of chitosan with DB18C6 (designed as Ch-DB18C6) were weighed exactly 0.1 g of extractant—dibenzo-18-crown ether 6 acid (Sigma-Aldrich, purity 98%) which further was dissolved in 25 mL nitrobenzene (99%, Carl Roth). Obtained solution was placed in contact with 1 g of chitosan (40 mesh, 90% deacetylation, molecular weight of 1.3 10^5^, purchased from Yuhuan Ocean Biology, China) in order to obtain a ratio extractant:suport = 0.1:1. Complete chitosan functionalization was obtained for a contact time of 24 h, after that wet adsorbent material was dried for 24 h at 323 K. Functionalized chitosan has been characterized by:

- scanning electron microscopy (SEM) coupled with energy dispersive X-ray spectroscopy (EDX) using a FEI Quanta FEG 250 scanning electron microscope;
- Fourier transform infrared spectroscopy using a Bruker Platinum ATR-QL Diamond spectrometer;
- Surface area was determined by using Brunauer, Emmet, Teller (BET) method, using an Quantachrome Nova 1200e analyzer.
- also was determined the point of zero charge (pZc)^22,23^.

pZc of synthesized adsorbent material has been determined by putting in contact 0.1 g Ch-DB18C6 adsorbent material with 25 mL KCl 0.1 M at 298 K, all systems were agitated at 200 rotations per minute using a thermostatic bath. KCl solutions pH has been adjusted between 2 and 12 by using NaOH solutions with concentrations between 0.05 and 2 N, or HNO3 solutions with concentrations between 0.05 and 2 N. Afterwards, all samples were filtered and solution pH has been determined using a pH-meter Mettler-Toledo SevenCompact.

In order to record the BET isotherms, all samples were degassed in vacuum at room temperature for minimum 24 h. For both samples the surface area has been obtained by using multi BET method, and the pore size and pore distribution has been determined from adsorption / desorption branches of the recorded isotherms by using Barrett, Joyner and Halenda (BJH) method. In order to determine the total pore volume was considered the last recorded point from the adsorption isotherm.

### Adsorption studies

In order to obtain information about adsorption process was studied the impact of various factors such as pH, temperature, contact time and initial concentration on the recovery of metallic ions on functionalized chitosan. pH influence is linked to the form of metallic ion into the solution and by the ionic form of the adsorbent functional groups.

In present paper has been studied the influence of solution pH on Pd(II), Ru(III) and Pt(IV) on produced functionalized chitosan. Studies were carried out by contacting 25 mL solution with 0.1 g of adsorbent material for a contact time of 2 h at 298 K, varying the pH from 0.5 to 10, for a metallic ions initial concentration of 10 mg L^−1^.

In order to establish the influence of contact time and temperature on the adsorption capacity of functionalized chitosan, 0.1 g of adsorbent material has been weighted over which 25 mL solution with metallic ions content (Pd(II), Ru(III), Pt(IV)) with concentration C0 = 10 mg L^−1^ was added. In order to establish time influence samples were mixed for different times (15, 30, 60, 120, 180 and 240 min) and at different temperatures (298 K, 308 K and 318 K). All samples were stirred at 200 rot min^−1^.

In order to establish the effect of initial concentration of metallic ions on adsorption capacity were prepared Pd (II), Ru (III) and Pt(IV) solution with concentrations 10, 25, 50, 75, 100, 150 and 175 mg L^−1^. All these solutions were prepared by dilution from stock one for Pd (5 wt. % Pd(II) chloride in 10 wt.% HCl) and Pt (Pt(IV) chloride 57.7%), respectively from Ru(III) chloride hydrate. Adsorption process has been performed at pH = 2 –98 K form 3 h. After adsorption residual concentration was measured using ICPOS technique performed on a 5100 VDV Agilent technologies with a double pass spray chamber and OneNeb nebulizer. For determination of each element were used the first two most intense spectra lines with 3 replicates and 5 s integration time for each.

### Kinetics and thermodinamics of adsorption: adsorption isotherm models

Equations used to describe adsorption equilibrium and adsorption kinetic are presented in Table 1.

**Table 1:** Equations used to describe adsorption equilibrium and adsorption kinetics.

Parameters	Equation	References
The adsorption capacity of the material	${{\text{q}}_{\text{e}}} = \frac{{\left( {{{\text{C}}_{0}} - {{\text{C}}_{\text{e}}}} \right) {\text{V}}}}{{\text{m}}}$ where q_e_, the maximum adsorption capacity (mg/g); C_0_, initial concentration of metallic ion in solution (mg L^−1^); C_e_, the equilibrium concentration of metallic ion in solution (mg L^−1^); V, volume of the aqueous solution with metallic ion content (L); m, mass of the adsorbent (g)	
Langmuir isotherm nonlinear expression	${{\text{q}}_{\text{e}}} = \frac{{{{\text{q}}_{\text{L}}}\,{{\text{K}}_{\text{L}}}\,{\text{C}}{}_{\text{e}}}}{{{1} + {{\text{K}}_{\text{L}}}\,{{\text{C}}_{\text{e}}}}}$ where *q*_*e*_, the maximum adsorption capacity (mg g^−1^); *C*_*e*_, the equilibrium concentration of metallic ion in solution (mg L^−1^); *q*_*L*_, Langmuir maximum adsorption capacity (mg g^−1^); *K*_*L*_, Langmuir constant	^24^
Freundlich isotherm nonlinear expression	${{\text{q}}_{\text{e}}} = {{\text{K}}_{\text{F}}}\,{\text{C}}_{\text{e}}^{{{1} \mathord{\left/ {\vphantom {{1} {{{\text{n}}_{\text{f}}}}}} \right. \kern-\nulldelimiterspace} {{{\text{n}}_{\text{f}}}}}}$ where *q*_*e*_, the maximum adsorption capacity (mg g^−1^); *C*_*e*_, the equilibrium concentration of metallic ion in solution (mg g^−1^); *K*_*F*_ *n*_*F*_, the characteristic constants that can be related to the relative adsorption capacity of the adsorbent and the intensity of adsorption	^28^
Sips isotherm nonlinear expression	${q_e} = \frac{{{q_s}\,{K_S}\,C_e^{{1 \mathord{\left/ {\vphantom {1 {n_S}}} \right. \kern-\nulldelimiterspace} {n_S}}}}}{{1 + {K_S}\,C_e^{{1 \mathord{\left/ {\vphantom {1 {n_S}}} \right. \kern-\nulldelimiterspace} {n_S}}}}}$ where *q*_*S*_, the maximum adsorption capacity (mg g^−1^); *K*_*S*_, constant related to the adsorption capacity of the adsorbent; *n*_*S*_, the heterogeneity factor	^29^
Pseudo-first order kinetic model (Lagergren)	${\text{ln}}\,\left( {{{\text{q}}_{\text{e}}} - {{\text{q}}_{\text{t}}}} \right) = {\text{ln}}{{\text{q}}_{\text{e}}} - {{\text{k}}_{1}} {\text{t}}$ where *q*_*e*_, equilibrium adsorption capacity (mg g^−1^); *q*_*t*_, adsorption capacity at a specific time – t (mg g^−1^); *k*_1_, pseudo-first order speed constant (min^−1^); *t*, contact time (min)	^30^
Pseudo-second order kinetic model (Ho and McKay)	$\frac{{\text{t}}}{{{{\text{q}}_{\text{t}}}}} = \frac{{1}}{{{{\text{k}}_{2}} {\text{q}}_{\text{e}}^{2}}} + \frac{{\text{t}}}{{{{\text{q}}_{\text{e}}}}}$ where *q*_*e*_, equilibrium absorption capacity (mg g^−1^); *q*_*t*_, adsorption capacity at a specific time—t (mg g^−1^); *k*_2_, pseudo-second order speed constant (g mg^−1^ min^−1^); *t*, contact time (min)	^31–33^

Determination of pseudo-first order speed constant and calculated adsorption capacity were determined from the linear dependence between ln(q_e_ − q_t_) versus t. When the experimental data were fitted using pseudo-second-order kinetic model, the parameters associated with this model were determined from the linear dependence between t/qt versus t. From line equation were determined the speed constant associated with pseudo-second-order model (k_2_) and pseudo-second-order model calculated adsorption capacity (q_e,calc_).

Further for considered metallic ions adsorption on Ch-DB18C6 were determined activation energy values from Arrhenius equation:

$$ \ln {\text{k}}_{2}  = \ln {\text{A}} - \frac{{{\text{E}}_{{\text{a}}} }}{{{\text{RT}}}} $$

where k_2_, speed constant (g min^−1^ mg^−1^); A, Arrhenius constant (g min mg^−1^); E_a_, activation energy (kJ mol^−1^); T, absolute temperature (K); R, ideal gas constant (8.314 J mol^−1^ k^−1^).

Determination of activation energy was possible by using the speed constant obtained from the pseudo-second-order model, which better fit the experimental data. Activation energy was calculated from the slope of linear dependence of lnk_2_ versus 1/T.

In order to establish if the metallic ions adsorption onto the adsorbent surface is a spontaneous process has been evaluated the value of free Gibbs energy from Gibbs–Helmholtz equation^34^:

$$ \Delta {\text{G}}^{ \circ }  = \Delta {\text{H}}^{ \circ }  - {\text{T}} \cdot \Delta {\text{S}}^{ \circ }  $$

where ΔG^0^, free Gibbs energy standard variation (kJ mol^−1^); ΔH^0^, enthalpy standard variation (kJ mol^−1^); ΔS^0^, entropy standard variation (J mol^−1^ k^−1^); T, absolute temperature (K).

Standard variations of enthalpy and entropy were evaluated from linear dependence of ln K_d_ versus 1/T (linear form of van’t Hoff equation), where K_d_ is the equilibrium constant, being calculated as ratio between equilibrium adsorption capacity (q_e_) and equilibrium concentration (C_e_).

### Sorption/desorption studies

For further practical application of such adsorbent materials it is important to evaluate their regenerative capacity. In order to optimize the cost of such processes, by reuse of adsorbent material, and to obtain good efficiency is necessary that the regenerative process occurs easy, whit a faster desorption of metallic ions in higher quantity.

In order to evaluate the maximum number of adsorption/desorption cycles, Pt(IV), Pd(II), Ru(III) ions were adsorbed and desorbed until the adsorption on used adsorbent material was not any more possible.

Desorption was performed by mixing 1 g of used CH-DB18C6 containing Pt(IV), Pd(II), and Ru(III) ions with 25 mL HCl 5%, obtained mixture has been shaken at 200 rotation per minute at room temperature for 3 h. After that, filtered adsorbent material has been rinsed with distilled water, and dried at room temperature. These steps were repeated several times until all metallic ions were irreversibly fixed onto the surface of adsorbent material. In this way has been establish the maximum number of usage cycles. Process efficiency was determined by counting the quantity of adsorbed / desorbed metallic ions.

### Recovery of precious metal ions

After adsorption the exhausted adsorbent material containing important quantities of Pd(II), Ru(III) and Pt(IV) have undergone a thermal decomposition at 600 K, for 240 min, into a controlled atmosphere oven (Nabertherm LHT407GN Furnaces). Produced sample has been analyzed using scanning electron microscopy (SEM) coupled with energy dispersive X-ray spectroscopy (EDX) (Quanta FEG 250 scanning electron microscope).

## Results and discussion

### Adsorbent characterization

**Figure 1:**
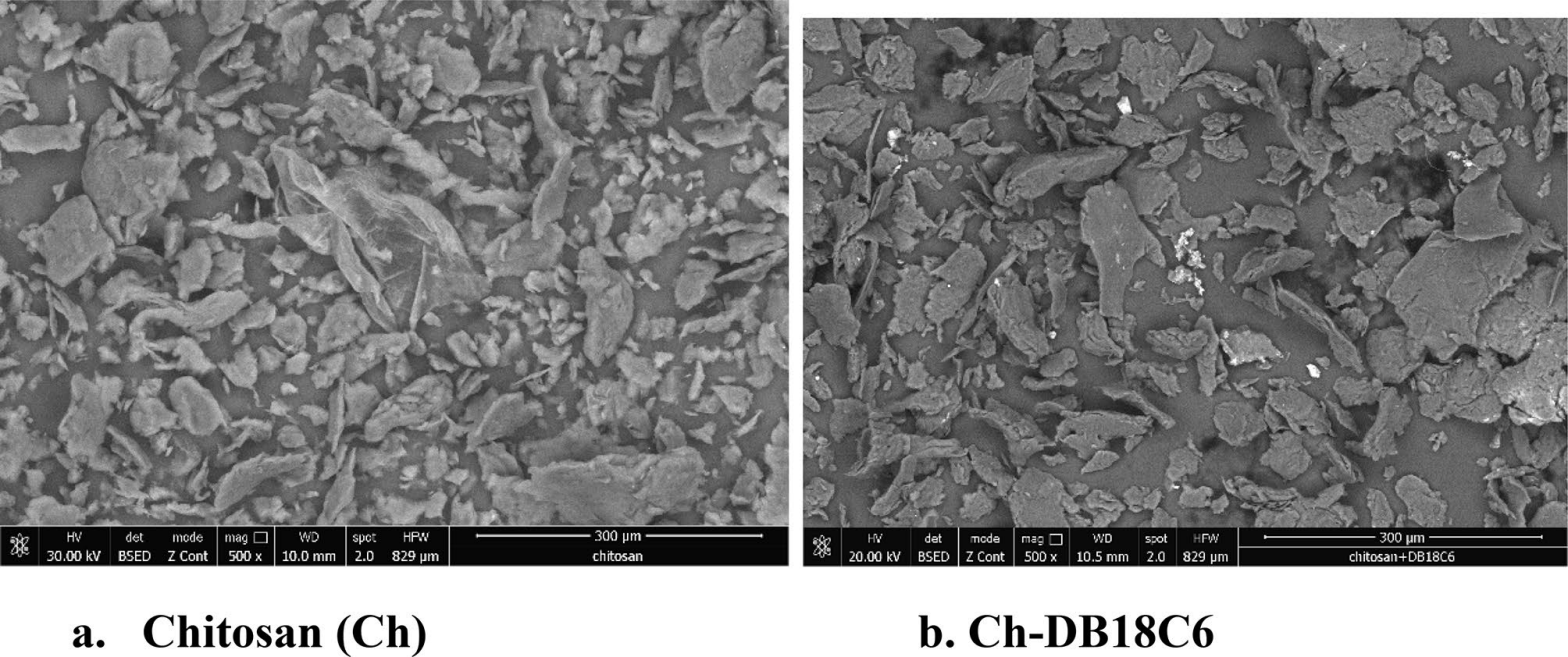
Scanning electron microscopy, SEM.

SEM technique has been used in order to analyze the chitosan surface before and after functionalization with DB18C6 (Fig. 1).

**Figure 2:**
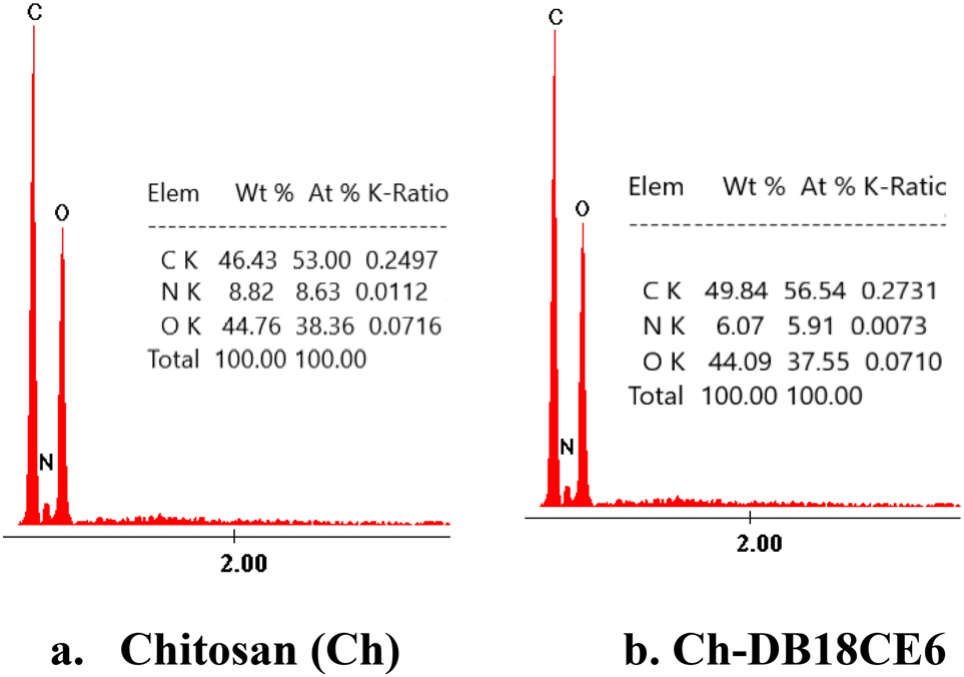
Energy-dispersive X-ray spectroscopy, EDX.

Analyzing images depicted in Fig. 1 can observe that after functionalization chitosan morphology was changed. Functionalization leads at certain increase of pore size. Further, functionalization was confirmed by recording the EDX spectra (depicted in Fig. 2). From these spectra can observe that after functionalization the C quantity increase from 46.46 at 49.84%, being in concordance with the data obtained from FT-IR. All these data prove the chitosan functionalization with DB18C6 crown ether.

**Figure 3:**
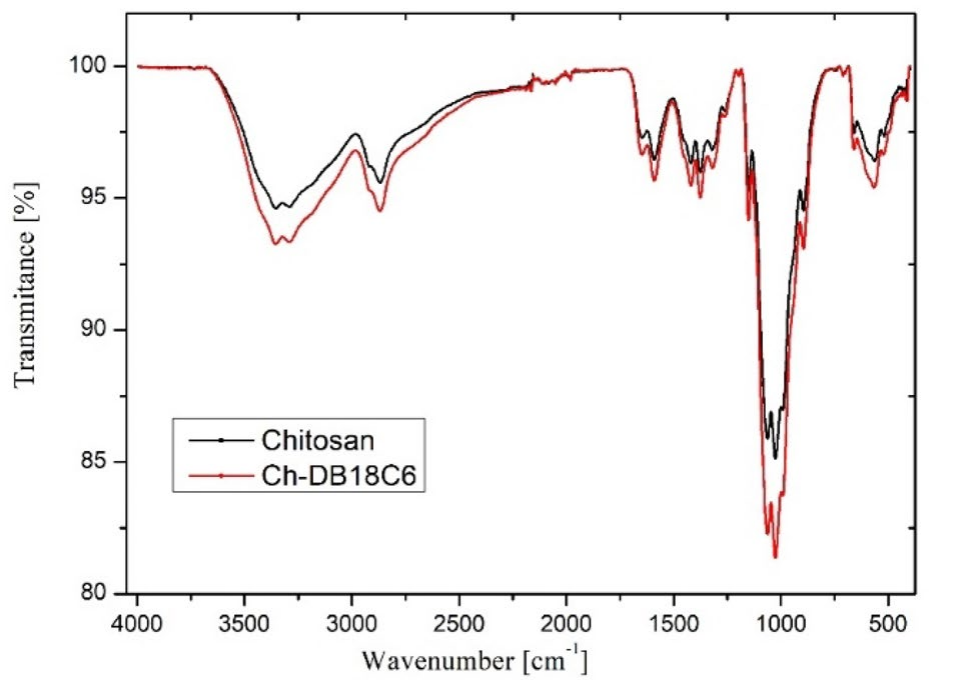
FT-IR spectra for Ch and Ch-DB18C6.

Further new produced adsorbent material has been characterized by recording the FT-IR spectra. Comparing the FT-IR spectra of chitosan with the spectra recorded for Ch-DB18C6 (Fig. 3) can observe that some modifications occur.

From comparison of the two FT-IR spectra can observe that the vibrations of new adsorbent material is similar with the chitosan’s, but more intense. Vibrations specific for N–H and O–H bonds appear at 3361 and 3291 cm^−1^. Adsorption bands located at 2910 and 2877 cm^−1^ can be attributed to the symmetric and asymmetric stretch of C–H bond, such bonds being specific to polysaccharides^35^. Presence of group N-acetyl residual it is confirmed by the vibrations located at 1645 and 1325 cm^−1^. Vibration located at 1153 cm^−1^ is associated asymmetric stretching of C–O–C bond and the bands located at 1066 and 1028 cm^−1^ are associated with the vibrations of C-O bonds^36–38^.

Presence of vibration at 3291 cm^−1^ is associated with the stretching of O–H bond and the one located at 2877 cm^−1^ is associated with symmetric and asymmetric stretching of C–H bond. Vibrations which appear at 1153 cm^−1^ are associated with the asymmetric stretching of C–O–C bonds. In functionalized chitosan vibrations located at 1066 and 1028 cm^−1^ present higher intensity and are associated with the stretching of C–O bond.

**Figure 4:**
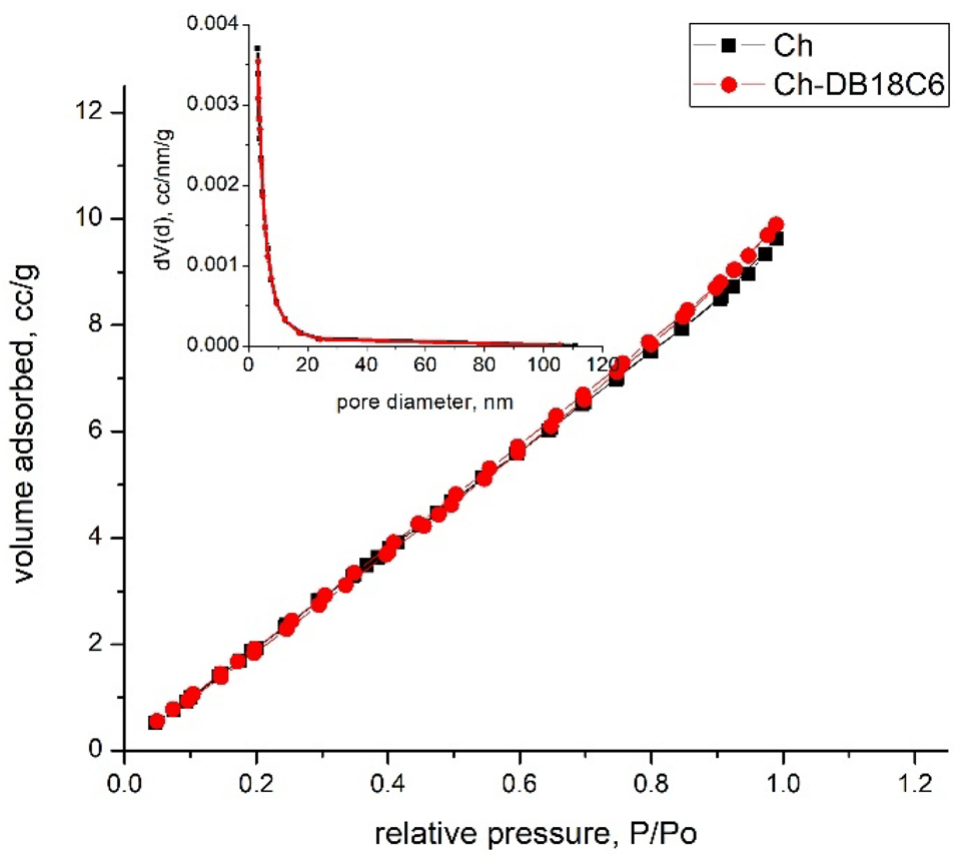
Adsorption isotherms.

Further, in order to prove the functionalization of chitosan with DB18C6 has been determined the specific surfaces for pure and functionalized chitosan by recording adsorption isotherms (Fig. 4).

Recorded isotherms are type II isotherms which are specific for nonporous and microporous adsorbents^39^, this being confirmed by the pore size distribution graph (inset figure). Based on recorded isotherms were calculated the specific parameters for pure and functionalized material (Table 2).

**Table 2:** Obtained textural parameters.

Sample name	Surface area (MultiBET), m2/g	pore size distribution (adsorption branch), nm	pore size distribution (desorption branch), nm	Total pore volume
Ch	15.25	3.414	3.062	1.493e−02 cc/g for pores smaller than 195.6 nm (diameter) at P/Po = 0.99007
Ch-DB18C6	14.44	3.436	3.220	1.534e−02 cc/g for pores smaller than 183.0 nm (diameter) at P/Po = 0.98938

From data presented in Table 2 can observe that the specific surface area is decreasing after functionalization, meaning that the BD18C6 extractant were included also into the Ch pores. This observation is in concordance with the data obtained from FT-IR proving in this way the functionalization of Ch with DB18C6.

**Figure 5:**
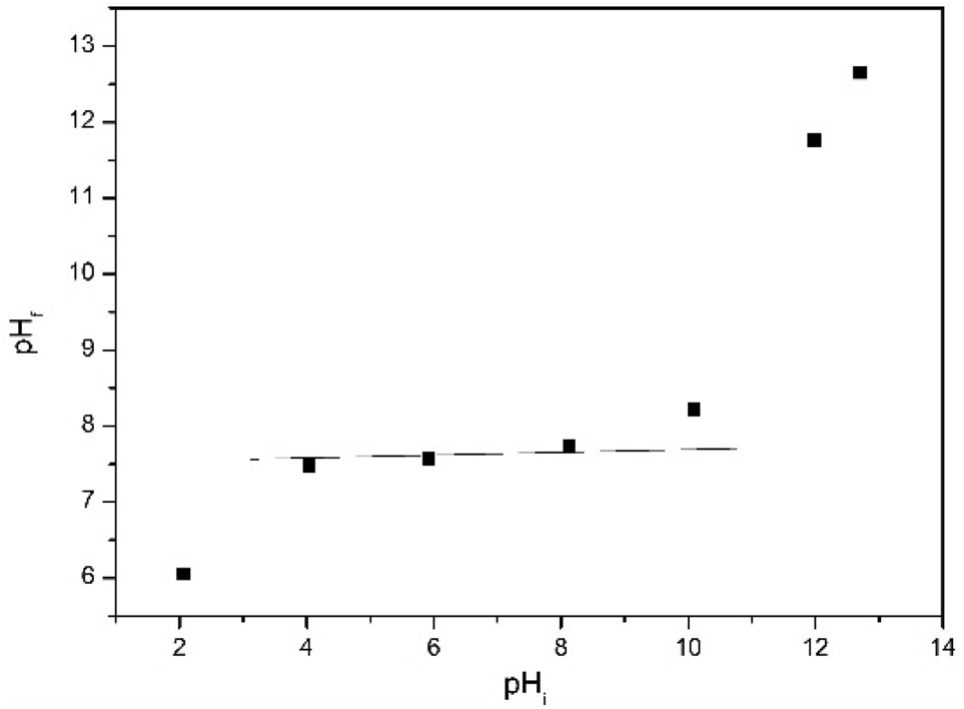
pZc effect for Ch-DB18C6.

It is known that for materials with adsorbent properties, is important to know their acid-basic properties for further usage. In this case, the existence of some potential at level of system interface due to the existence of H^+^/HO^−^ ions can be expressed in terms of pH and is called zero charge point. For determination of pHpZc has been depicted the dependence between final value of pH (pHf) and initial value (pHi) (Fig. 5).

Analyzing data presented in Fig. 4 can observe that for each value of initial pH located between 2 and 10 new produced material present buffering capabilities. Ch-DB18C6 present a value of 7.5 for pZc, located into the pH interval 4 to 10, meaning that such material can be used for adsorption processes conducted between these two values of pH.

### Effect of pH

**Figure 6:**
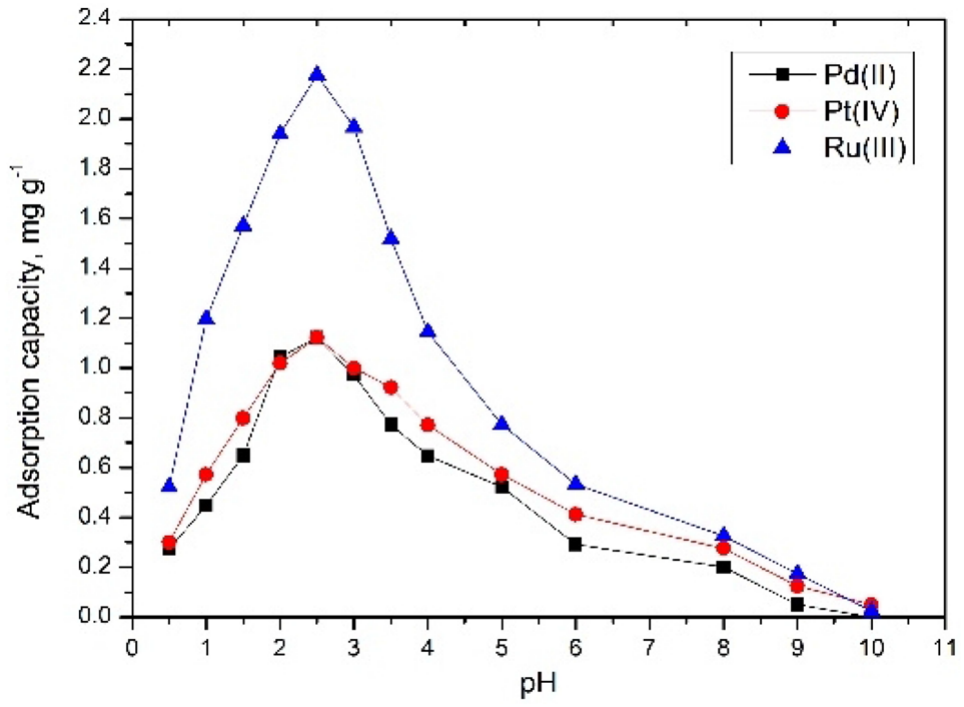
pH effect.

pH effect on studied metallic ions adsorption is presented in Fig. 6. Analyzing obtained data can observe that for all three studied metallic ions the maximum adsorption capacity increases as long as solution pH is kept lower than 3. Further increase of pH has as effect a rapid decrease of maximum adsorption capacity. This behavior can be explained if we are taking into account that at lower pH, HCl concentration into the solution is high enough in order to favor formation of chloro-anionic species (${\text{PtCl}}_{6}^{2-}$, ${\text{PdCl}}_{4}^{2-}$, ${\text{RuCl}}_{4}^{-}$ )^40^.

These ionic species can be adsorbed onto the surface of –NH_2_ groups found on chitosan surface^13^ and, in same time, these species be complexed inside of the ring of crow ether^41^ which explain its good adsorption capacity.

Decrease of adsorption capacity at pH higher than 3 can be explained if we consider the lower concentration of chloride ions into the solution which lead at some decrease into the concentration of absorbable species.

All these observations are in consensus with data found in the literature, which confirm that the adsorption of studied metallic ions better occur at low pH, when the competitive adsorption is reduced^42–44^.

### Effect of contact time and temperature: kinetics and thermodynamic studies

**Figure 7:**
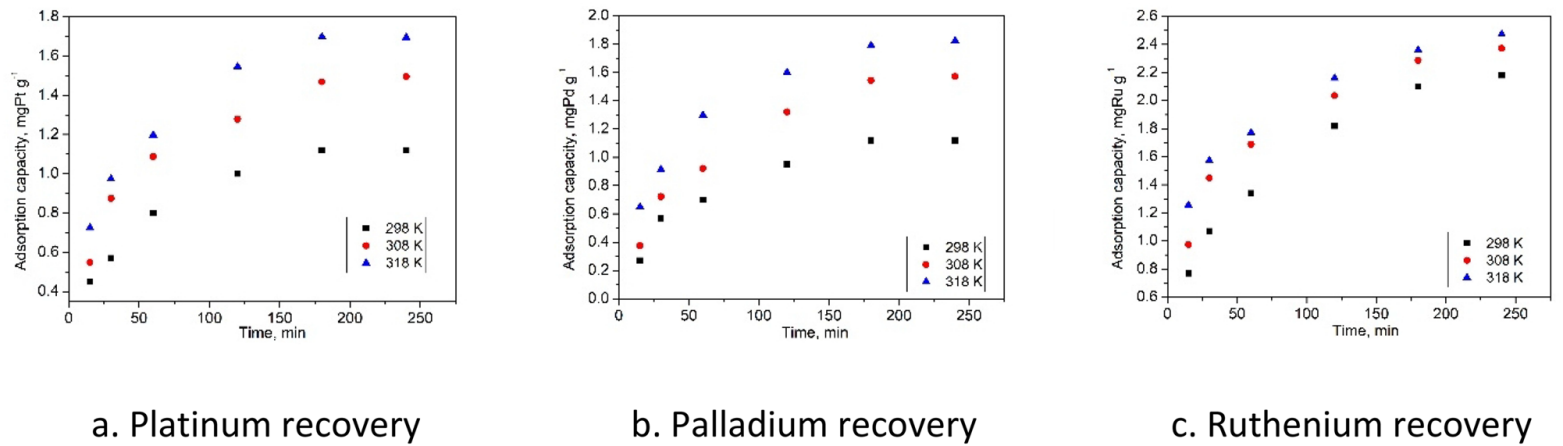
Contact time at different temperatures.

Other important parameter for adsorptive processes is represented by the contact time at different temperatures. Contact time influence for adsorption of Pd(II), Ru(III) and Pt(IV) ions is presented in Fig. 7.

**Figure 8:**
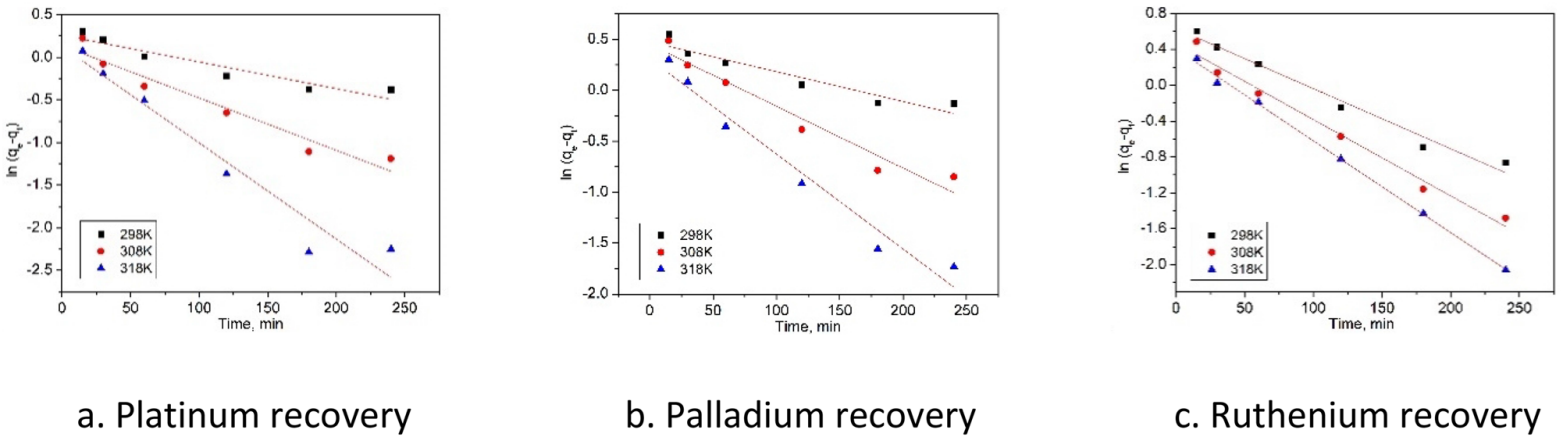
Preudo-first-order kinetic model of the Pt(IV), Pd(II) şi Ru(III) recovery by Ch-DB18C6.

**Figure 9:**
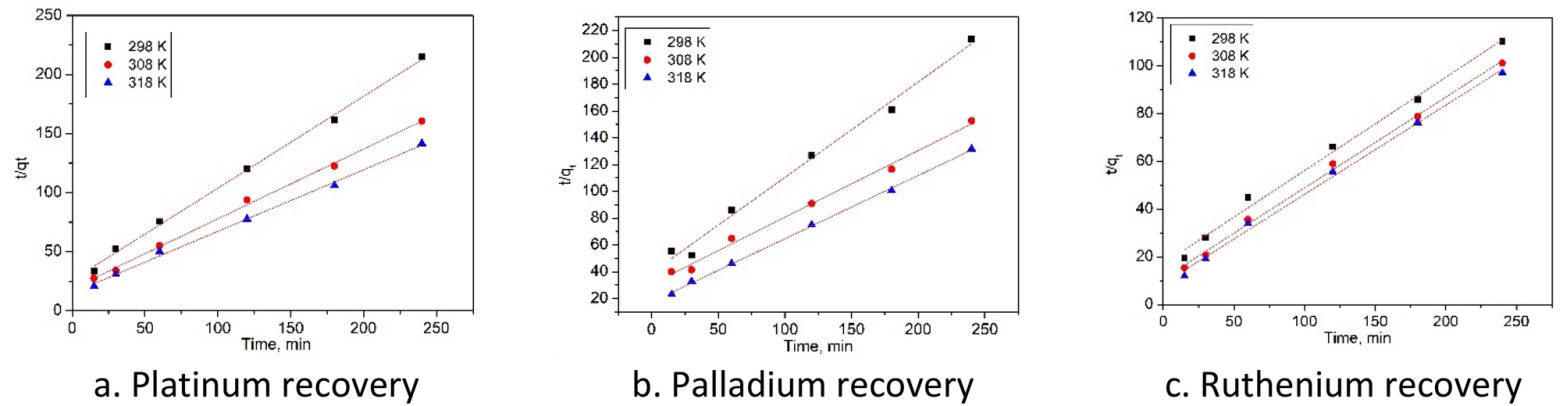
Pseudo-second-order kinetic model of the Pt(IV), Pd(II) şi Ru(III) recovery by Ch-DB18C6.

Obtained experimental data proved that the contact time increase leads at increase of maximum adsorption capacity for all three metallic ions. Maximum adsorption capacity was reached when the contact time was around 180 min, further increase of contact time having no notable influence on maximum adsorption capacity. In order to increase the precision for any further experiment the contact time was set at 180 min. Further for establishing the mechanism of the studied adsorption process, obtained experimental data were modeled using two different kinetic models. In Fig. 8 are depicted the data obtained after modelling the experimental results using pseudo-first-order kinetic model and in Fig. 9 are depicted data obtained after modelling experimental results with pseudo-second-order kinetic model. Based on these models were obtained kinetic parameters which are presented in Table 3.

**Table 3:** Kinetic parameters for adsorption of Pt(IV), Pd(II) and Ru(III) onto Ch-DB18C6 material.

Temp (K)	q_e,exp_ (mg g^−1^)	Pseudo-first order kinetic model				Pseudo-second order kinetic model			
		q_e,calc_ (mg g^−1^)	k_1_ (min^−1^)	R^2^	χ^2^	q_e,calc_ (mg g^−1^)	k_2_ (min^−1^ mg^−1^ g)	R^2^	χ^2^
Pt(IV)									
298	0.99	1.29	0.0031	0.9022	0.32	1.28	0.06357	0.9974	0.017
308	1.27	1.14	0.0061	0.9446	0.41	1.69	0.15282	0.9978	0.022
318	1.54	1.13	0.0113	0.9461	0.44	1.91	0.24378	0.9973	0.012
Pd(II)									
298	0.95	1.61	0.0029	0.9053	0.21	1.40	0.05022	0.9918	0.027
308	1.32	1.57	0.006	0.952	0.28	2.01	0.13039	0.9939	0.031
318	1.60	1.35	0.0093	0.9676	0.23	2.11	0.25742	0.9990	0.033
Ru(III)									
298	1.81	1.88	0.0067	0.9796	0.23	2.10	0.383667	0.9936	0.021
308	2.04	1.59	0.0085	0.984	0.82	2.12	0.636909	0.9975	0.037
318	2.16	1.49	0.0102	0.9982	0.28	2.37	0.923943	0.9966	0.011

In order to validate the model which, describe studied adsorption process, were analyzed the values of regression coefficient and of the maximum adsorption capacities. When the regression coefficient value is closer to 1 can assume that the model is better described by the kinetic mechanism for the studied adsorption process. Based on data presented in Table 2 can observe that for all studied adsorptions, process kinetic is better described by pseudo-second-order model.

Also, can observe that the maximum adsorption capacity estimated based on this model is closer to the experimental maximum adsorption capacity.

These observations can indicate that the adsorptions of Pd(II), Ru(III) and Pt(IV) ions on produced adsorbent is a process similar to a chemical adsorption. Can predict that such adsorptions involve certain valence bonds between free electrons of metallic ions complex anions and the surface of Ch-DB18C6 adsorbent material^3^.

Adsorption of studied metallic ions onto the Ch-DB18C6 it is a two stage process: (i) in first stage occurs an instantaneous adsorption on adsorbent surface, (ii) second stage can be a trapped adsorption when the limiting step is the particle diffusion^45,46^.

**Figure 10:**
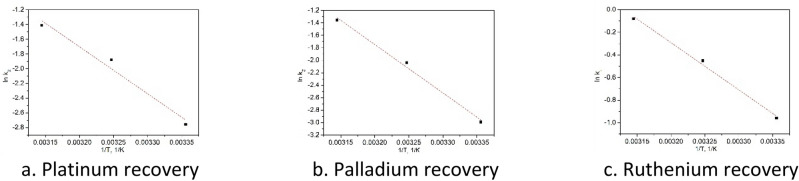
lnK_2_ versus 1/T for adsorptions onto Ch-DB18C6.

In order to confirm the nature of studied adsorption processes were evaluated the values of activation energy using Arrhenius equation (data are depicted in Fig. 10). From linear dependence between ln k_2_ and 1/T were calculated the values of E_a_, presented in Table 4.

**Table 4:** Activation energy.

Metallic ion	Activation energy E_a_ kJ mol^−1^	R^2^
Pt(IV)	53.1	0.9960
Pd(II)	64.5	0.9940
Ru(III)	40.6	0.9952

Values of E_a_ obtained for Pd(II), Ru(III) and Pt(IV) adsorptions are greater than 40 kJ mol^−1^ which confirm that these adsorption processes are chemical one^47^.

Further, has been investigated temperature effect on Pd(II), Ru(III), Pt(IV) adsorptions by conducting experiments at three different temperatures (298, 303 and 318 K). From obtained experimental data can observe that temperature increase has a beneficial effect for all three studied adsorption processes.

**Figure 11:**
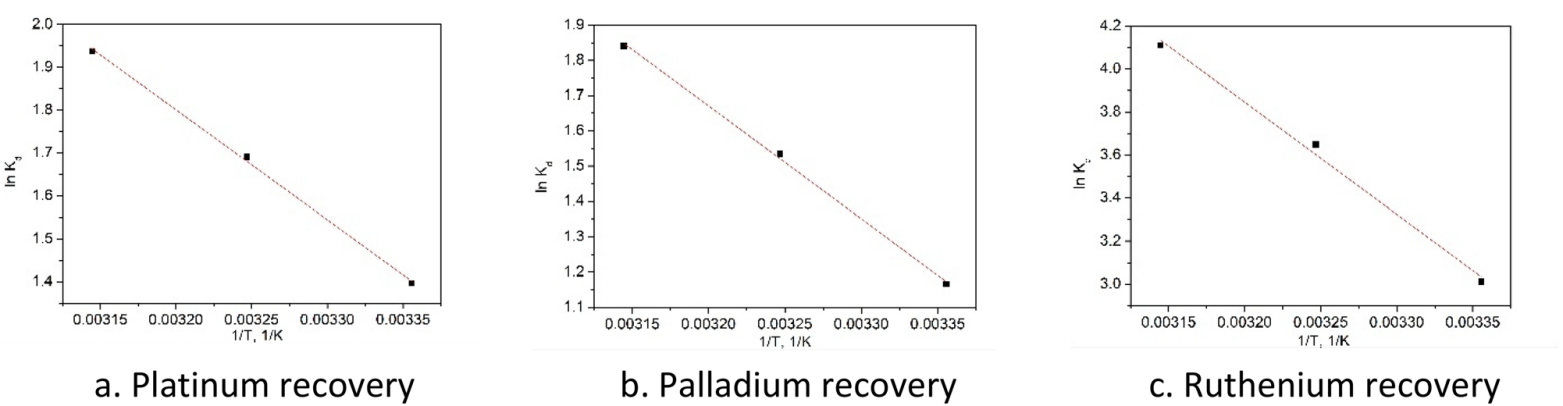
ln K_d_ versus 1/T for adsorptions on Ch-DB18C6.

Based on obtained experimental data, were evaluated the values of free Gibbs energy variation, enthalpy variation and entropy variation from linear form of van’t Hoff equation (Fig. 11). Values of thermodynamic parameters were estimated from linear dependence between ln k_d_ and 1/T, (Table 5).

**Table 5:** Thermodynamic parameters for the adsorption of Pt (IV), Pd (II) and Ru(III) onto Ch-DB18C6 material.

Temp (K)	Pt(IV)			Pd(II)			Ru(III)		
	ΔG^o^ (kJ mol^−1^)	ΔH^o^ (kJ mol^−1^)	ΔS^o^ (kJ mol ^−1^ K^−1^)	ΔG^o^ (kJ mol^−1^)	ΔH^o^ (kJ mol^−1^)	ΔS^o^ (kJ mol^−1^ K^−1^)	ΔG^o^ (kJ mol^−1^)	ΔH^o^ (kJ mol^−1^)	ΔS^o^ (kJ mol^−1^ K^−1^)
298	− 3.81	21.34	83.3	− 2.84	26.61	99.1	− 7.07	43.3	170.6
308	− 4.62			− 3.37			− 9.60		
318	− 5.44			− 4.90			− 10.14		

Analyzing data presented in Table 4 can observe that for all three studied processes the values obtained for free Gibbs energy are negative which suggest that all adsorption processes are spontaneous. It was also observed that the value of ΔG^0^ decreases with temperature increase, meaning that the temperature favors adsorption processes, leading at increase of maximum adsorption capacity. Positive values of ΔH° and ΔS° confirm that studied adsorption processes are endothermic.

### Effect of initial concentration of metal ions: adsorption isotherms

**Figure 12:**
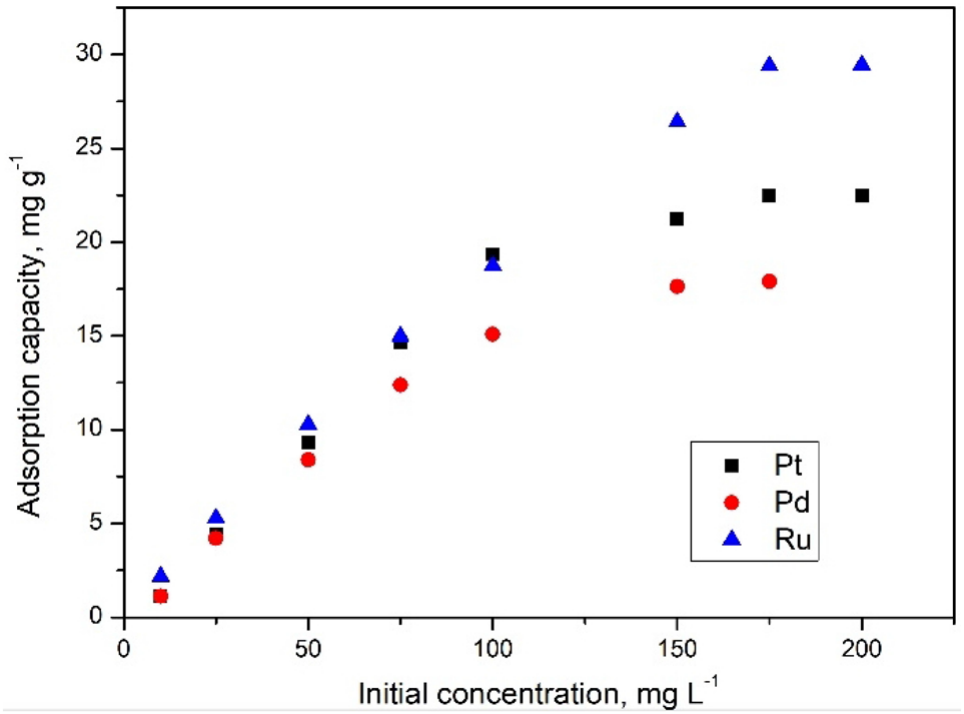
Effect of initial concentration of Pt(IV), Pd(II) and Ru(III) onto adsorption process.

In order to better understand studied adsorption processes were evaluated the influences of initial concentrations on the maximum adsorption capacities (data depicted in Fig. 12).

Obtained experimental data proved that the maximum adsorption capacities increase with increases of initial concentrations until a maximum value is reached. When maximum adsorption capacities were obtained can conclude that the adsorbent material has been saturated and any further increase into the initial concentrations has no further effect on adsorption capacities. During experiments has been obtained a maximum adsorption capacity of: 22.4 mg g-1 for Pt(IV) initial concentration of 175 mg L^−1^, 17.6 mg g^−1^ for Pd(II) initial concentration of 150 mg L^−1^, and 30.4 mg g^−1^ for Ru(III) initial concentration of 175 mg L^−1^.

**Figure 13:**
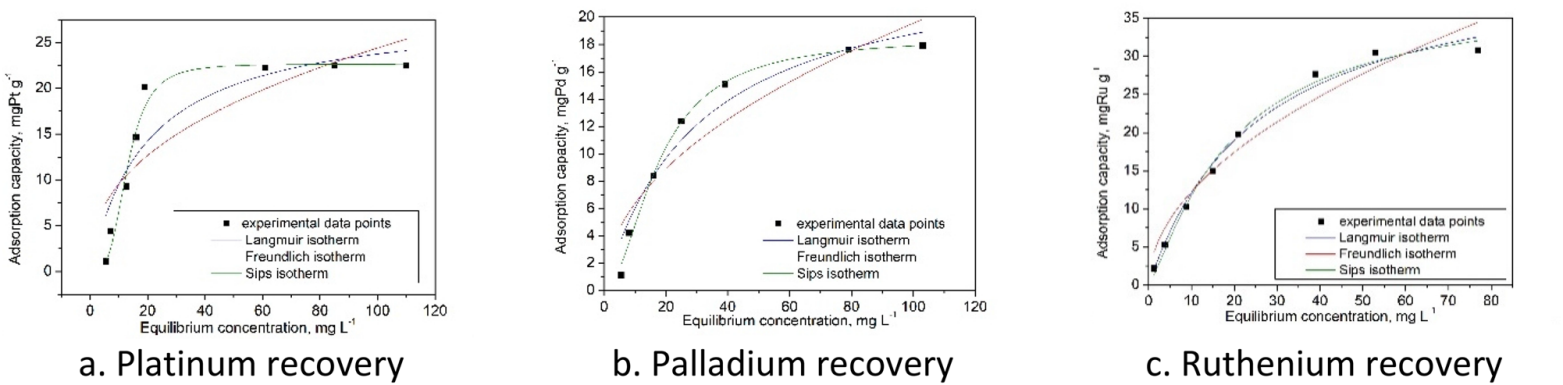
Adsorption isotherms.

In order to understand and describe adsorption mechanism of studied ions onto the new produced adsorbent material experimental data were fitted using Freundlich, Langmuir and Sips models (Fig. 12). Based on data presented in Fig. 13 were determined the parameters specific for each used adsorption isotherms (Table 6).

**Table 6:** Langmuir, Freundlich and Sips isotherm parameters for Pt(IV), Pd(II) and Ru(III) ions adsorption onto Ch-DB18C6 material.

Metal ions	q_m,exp_ (mg g^−1^)	Langmuir isotherm			Freundlich isotherm			Sips isotherm			
		q_L_ (mg g^−1^)	K_L_	R^2^	K_F_ (mg g^−1^)	1/n_F_	R^2^	K_s_	q_s_ (mg g^−1^)	1/n_s_	R^2^
Pt(IV)	**22.4**	28.4	0.050	0.8928	3.7	0.4	0.7897	0.0026	**22.6**	0.8	**0.9901**
Pd(II)	**17.6**	20.3	0.051	0.8968	2.8	0.3	0.8631	0.0013	**19.2**	0.6	**0.9904**
Ru(III)	**30.4**	40.4	0.040	0.9887	3.8	0.5	0.9450	0.0270	**33.1**	0.3	**0.9908**

Analyzing data presented in Table 6 can observe that the higher regression coefficient has been obtained when the experimental data were fitted using Sips model (regression coefficient was ~ 0.99). Based on data presented in Table 5 can conclude that the Sips model accurately describes the Pd, Ru, and Pt adsorption on prepared material.

In Table 7 are presented several similar materials used for recovery of PGM’s ions by adsorption from different solutions.

**Table 7:** Adsorption capacities of some adsorbents cited in the literature.

Adsorbent	Adsorption capacities, mg g^−1^			Reference
	Pt(IV)	Pd(II)	Ru(III)	
Fe_3_O_4_ nano-particles	13.3	10.96	–	^40^
3-(8-Quinolinylazo)-4-hydroxybenzoic acid modified nanometer-sized alumina 17.7 7.6 [17] (E,E,E)-1-[(4-methylphenyl)sulfonyl]-6-[(2-trimethylsilylethyl) sulfonyl]-11-[(4-vinylphenyl) sulfonyl)]-1,6,11-triazacyclopentadeca-3,8,13-triene functionalized polystyrene	–	7.6	–	^48^
Amidinothioureido-silica gel	–	15	–	^48^
Clinoptilolite	–	–	15	^49^
Activated charcoal	–	–	12	^50^
Ch-DB18C6	17.6	22.6	30.4	Present paper

### Recovery precious metal ions

After extraction of metallic ions from aqueous solutions, exhausted Ch-DB18C6 adsorbent material was calcinated at 873 K in controlled atmosphere in order to recover metallic particles. This treatment was made by heating samples with a speed of 5 K per minute and kept at 873 K for minimum 240 min.

**Figure 14:**
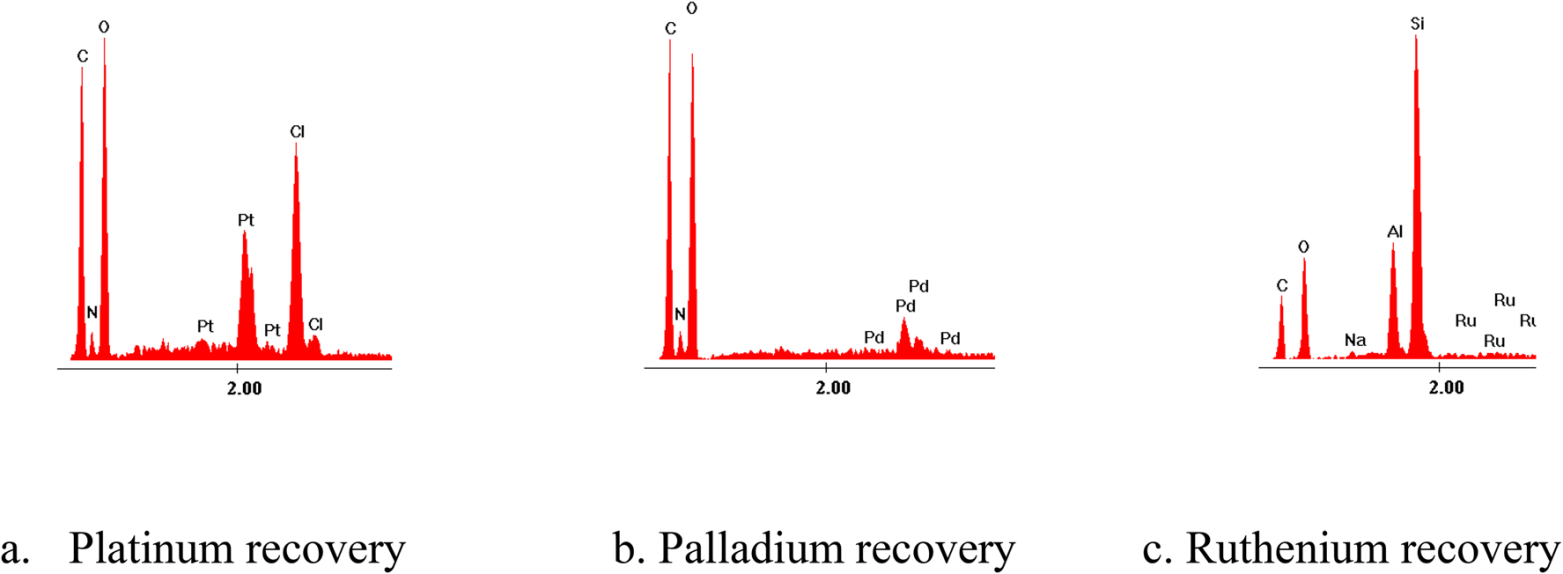
Energy dispersive X-ray analysis, EDX after recovery metallic ions.

After calcination obtained samples were characterized by recording EDX spectra (Fig. 14).

Based on EDX spectra presented in Fig. 14 can observe the presence of Pd, Ru and Pt specific peaks into the ash obtained after calcination. Also, in these EDX spectra’s were identified the peaks specific for ash components. Based on these observations can conclude that the desired metals can be recovered from exhausted adsorbent material.

### Sorption/desorption studies

**Figure 15:**
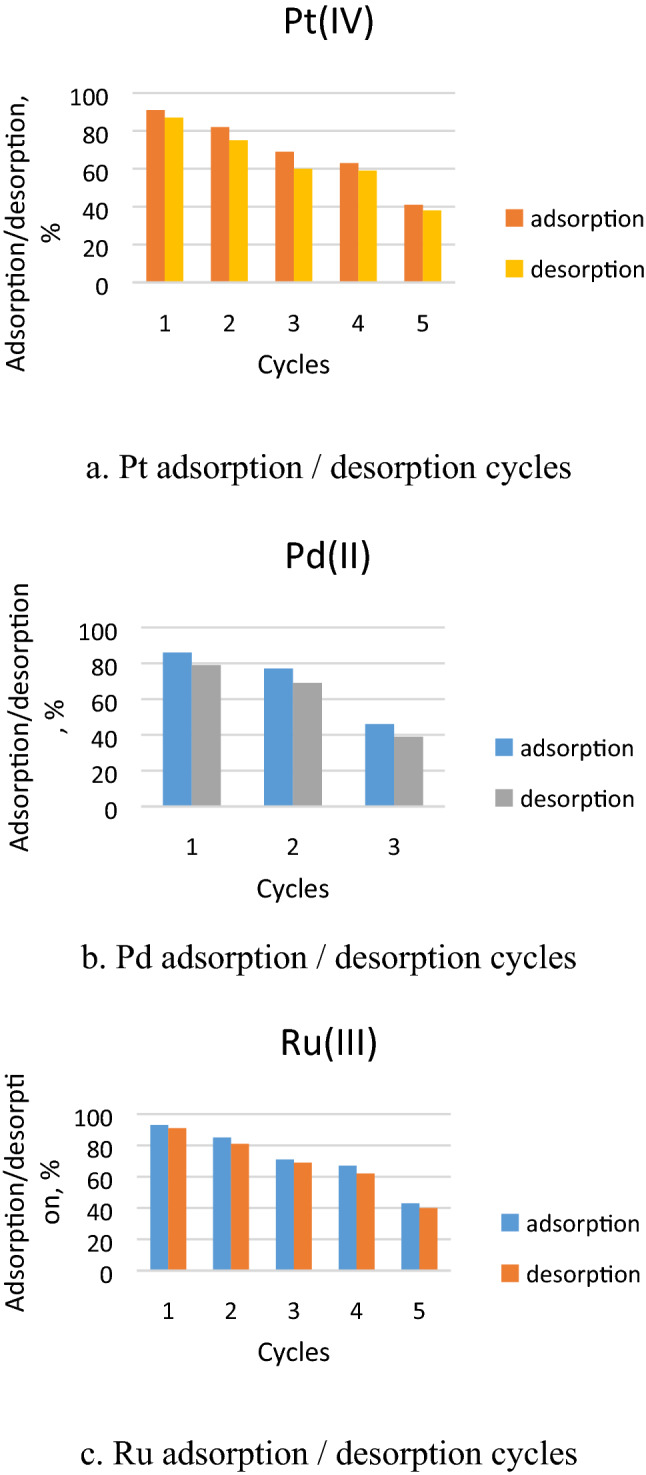
Adsorption/desorption cycles obtained for CH-DB18C6 adsorbent material.

In present paper was investigated the possibility to reuse the Ch-DB18C6 material used adsorbent by establishing the sorption/desorption cycles number. Sorption / desorption studies were repeated with significant results for 5 times for Pt (IV) and Ru (III) and 3 times for Pd(II) as can be observed from data depicted in Fig. 15.

## Conclusions

Present study confirms that DB18C6 crown ether was successful used as extractant for functionalization of chitosan by SIR method. Obtained adsorbent material has been used for further recovery of PGM’s. Chitosan functionalization has been evidenced by using physic-chemical characterization of new produced adsorbent material. In this context were recorded FT-IR and EDX spectra, SEM micrographs, and was determined the value of pHpzc, which proved the preparation of desired adsorbent materials.

Obtained experimental results proved that the PMG’s ions adsorption depends on solution pH, contact time, temperature and initial concentration of the metallic ions into the solution.

From obtained experimental data was observed that CH-DB18C6 adsorbent presents the highest adsorption capacity for Ru(III) ions. For all studied systems were performed kinetic, thermodynamic, and equilibrium studies in order to establish metallic ions adsorption mechanism.

Based on obtained experimental data was established that the studied adsorptions are better described by pseudo-second-order model. Concomitant obtained experimental data are well fitted by Sips isotherm for all studied metallic ions. Based on obtained information can conclude that the PGM’s recovery by adsorption is an endothermal, spontaneous process influenced by temperature. Starting from exhausted adsorbent material it is possible to recover pure metals by thermal decomposition into controlled atmosphere.
